# Crystal structures of highly specific phosphinic tripeptide enantiomers in complex with the angiotensin‐I converting enzyme

**DOI:** 10.1111/febs.12660

**Published:** 2013-12-24

**Authors:** Geoffrey Masuyer, Mohd Akif, Bertrand Czarny, Fabrice Beau, Sylva L. U. Schwager, Edward D. Sturrock, R. Elwyn Isaac, Vincent Dive, K. Ravi Acharya

**Affiliations:** ^1^ Department of Biology and BiochemistryUniversity of BathUK; ^2^ Service d'Ingénierie Moléculaire des ProtéinesCEAiBiTecSGif‐sur‐YvetteFrance; ^3^ Division of Medical BiochemistryInstitute of Infectious Disease and Molecular MedicineUniversity of Cape TownSouth Africa; ^4^ School of BiologyUniversity of LeedsUK; ^5^ Department of BiochemistryUniversity of HyderabadIndia

**Keywords:** angiotensin‐I converting enzyme (ACE), *Drosophila melanogaster*, inhibitor binding, stereochemistry, X‐ray crystallography, zinc metallopeptidase

## Abstract

Human somatic angiotensin‐I converting enzyme (ACE) is a zinc‐dependent dipeptidyl carboxypeptidase and a central component of the renin angiotensin aldosterone system (RAAS). Its involvement in the modulation of physiological actions of peptide hormones has positioned ACE as an important therapeutic target for the treatment of hypertension and cardiovascular disorders. Here, we report the crystal structures of the two catalytic domains of human ACE (N‐ and C‐) in complex with FI, the *S* enantiomer of the phosphinic ACE/ECE‐1 (endothelin converting enzyme) dual inhibitor FII, to a resolution of 1.91 and 1.85 Å, respectively. In addition, we have determined the structure of AnCE (an ACE homologue from *Drosophila melanogaster*) in complex with both isomers. The inhibitor FI (*S* configuration) can adapt to the active site of ACE catalytic domains and shows key differences in its binding mechanism mostly through the reorientation of the isoxazole phenyl side group at the P_1_′ position compared with FII (*R* configuration). Differences in binding are also observed between FI and FII in complex with AnCE. Thus, the new structures of the ACE–inhibitor complexes presented here provide useful information for further exploration of ACE inhibitor pharmacophores involving phosphinic peptides and illustrate the role of chirality in enhancing drug specificity.

**Database:**

Structural data are available in the Protein Data Bank databases under accession numbers 4ca5, 4ca6, 4ca7, 4ca8.

**Abbreviations**
ACE: angiotensin‐I converting enzyme
AnCE: ACE homologue from *Drosophila melanogaster*
ECE‐1: endothelin converting enzyme‐1

## Introduction

Human angiotensin‐I converting enzyme (ACE, EC 3.4.15.1) is a central component of the renin angiotensin aldosterone system (RAAS), which controls blood pressure, electrolyte homeostasis, renal and vascular function and myocardial remodelling (for reviews see 1). ACE is a membrane‐bound zinc metalloprotease and a member of the gluzincin family. Its dipeptidyl carboxypeptidase activity cleaves many peptides *in vivo*, the major ones being angiotensin II (vasopressor octapeptide) 6 and bradykinin (vasodepressor nonapeptide) 7. The activation of angiotensin I (the inactive decapeptide) to angiotensin II and the inactivation of bradykinin lead to vasoconstriction. The current ACE inhibitors widely used in the treatment of hypertension, congestive heart failure and diabetic nephropathy 9 are designed based on the principle that suppression of angiotensin II formation and bradykinin degradation is clinically important.

ACE is found as two isoforms in humans: somatic ACE, which is composed of the homologous catalytic domains N‐ACE and C‐ACE (which share ~ 60% amino acid sequence identity 11) and testis ACE (tACE) which is a single domain protein and, apart from a short peptide sequence at the N‐terminus, is identical to C‐ACE 12. Both domains can cleave angiotensin I; however, C‐ACE has been shown to be sufficient for maintaining the regulation of blood pressure *in vivo* 13 and is considered the dominant site for angiotensin II production. In contrast N‐ACE plays a specific role in the regulation of hematopoietic stem cell differentiation and proliferation through hydrolysis of the anti‐fibrotic natural hemoregulatory peptide AcSDKP (*N*‐acetyl‐seryl‐aspartyl‐lysyl‐proline) 14. Furthermore, the two domains present distinctive physicochemical properties in terms of thermostability 15, resistance to proteolysis 16, chloride ion dependence 17 and substrate specificity 14.

A number of commercially available ACE inhibitors including captopril, lisinopril, enalapril and perindopril were designed in the 1970s, based on the three‐dimensional structure of carboxypeptidase and chemical knowledge of known zinc metalloproteases 21. However, it has now been established that prolonged clinical usage of these inhibitors can cause undesirable side effects such as persistent dry cough, loss of taste and angioedema due to elevated levels of bradykinin 23. In order to reduce the side effects during ACE inhibitor therapy, it is desirable to block the conversion of angiotensin I to angiotensin II by ACE (known to be performed *in vivo* mainly by C‐ACE) without interfering with the degradation of bradykinin 2. Hence the design of novel, second generation ACE inhibitors that selectively target C‐ACE for the treatment of hypertension and cardiovascular diseases remains a clinically important goal. Research in this direction has been boosted by the availability since 2003 of high resolution molecular structures of testis ACE (C‐ACE) in complex with known inhibitors and their derivatives 26.

A major advance in understanding the role of individual catalytic domains of ACE at the molecular level has come about with the development of domain‐specific phosphinic peptide based inhibitors 31. These peptides were designed to take advantage of the weaker coordinating power of the phosphate toward the catalytic zinc ion compared with clinically used ACE inhibitors and through optimized binding in the inner core of the molecule involving the catalytic site. Two important phosphinic peptides RXP407 (N‐ACE selective inhibitor 32) and RXPA380 (C‐ACE selective inhibitor 33) have been described by Dive *et al*. They also demonstrated that RXP407 upregulated AcSDKP metabolism by increasing the plasma levels (4–6‐fold) with little effect on blood pressure regulation 34. On the other hand, RXPA380 inhibition of C‐ACE did not prevent the degradation of bradykinin which is performed by N‐ACE 33.

**Figure 1:**
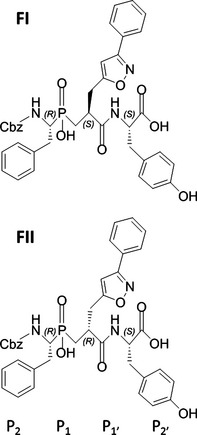
Structure of the inhibitor enantiomers FI and FII.

Further impetus to the improved design of phosphinic tripeptide inhibitors was recently provided by the design of an ACE/ECE‐1 (endothelin converting enzyme‐1) ‘dual inhibitor’, FII (Fig. 1) 35. Targeting both ACE and ECE‐1, FII lowers plasma concentrations of angiotensin I and endothelin 1, the two most potent vasoconstrictive peptides, without increasing bradykinin levels 36. FII is of particular interest since it presents an unusual *R* configuration of the P_1_′ moiety. Its stereoisomer FI, on the other hand, possesses an *S* configuration which makes it a less specific inhibitor, showing potent activity not only on ACE and ECE‐1 but also on neprilysin and MMP‐13 35.

The issue of chirality in drug design has been a growing concern and has resulted in most of the recently approved drugs being single enantiomers 37. Racemic products often rely on the activity of one enantiomer while the diastereomeric molecule may present unwanted effects 38. The difference in specificity between FI and FII therefore provides a good example of the importance of chirality in drug development.

The ACE homologue AnCE, from an invertebrate, *Drosophila melanogaster,* has been studied in much detail, both at the biochemical and structural levels. AnCE is a single domain protein and was reported to have biochemical resemblance to C‐ACE 39. In addition, the three‐dimensional structures of native AnCE and its complexes with ACE inhibitors have firmly established the high degree of conservation in the active site 41. These structures have been useful in understanding the behaviour of the ‘chemical space’ at the active site of ACE and its homologues.

In order to investigate the structural basis of the specific phosphinic tripeptide enantiomer binding to ACE homologues, we have co‐crystallized FI (*S*) with human ACE (N‐ and C‐ACE) and the structures were compared with the previously reported structures of FII (*R*) with human ACE (N‐ and C‐ACE). Furthermore, the structures of complexes FI (*S*) and FII (*R*) with AnCE were determined at high resolution. The structural information highlights key differences in the binding of FI and FII, and furthers our understanding of the rationale behind the specificity of FII as a dual inhibitor targeting ACE and ECE‐1. This study will help to further the design of molecules with enhanced specificity and potency towards multiple selected enzymes.

## Results

### Overall structure of ACE in complex with the inhibitors

The structures of C‐ACE, N‐ACE and AnCE in complexes with the dual inhibitors were refined between 1.8 and 2.0 Å resolution. All three proteins crystallized in the same space group as their native counterpart as described previously 26. In all four cases the catalytic zinc ion was present at the active site along with one inhibitor molecule. The *N*‐linked glycans were seen for each protein and included in the final coordinates. Binding of the inhibitor did not produce any noticeable conformational change in any of the proteins studied. The crystallographic details of the complex structures are presented in Table 1.

**Table 1:** Data collection and refinement statistics. Values in parentheses are for the last resolution shell. ^a^ *R*_symm_ = Σ*h*Σ_*i*_[|*I*_*i*_(*h*) − 〈*I*(*h*)〉|/Σ*h*Σ_*i*_ *I*_*i*_(*h*)], where *I*_*i*_ is the *i*th measurement and 〈*I*(*h*)〉 is the weighted mean of all the measurements of *I*(*h*). ^b^ *R*_cryst_ = Σ*h*|*F*_o_ − *F*_c_|/Σ*hF*_o_, where *F*_o_ and *F*_c_ are observed and calculated structure factor amplitudes of reflection *h*, respectively. ^c^ *R*_free_ is equal to *R*_cryst_ for a randomly selected 5% subset of reflections. The two values in the *B*‐factor analysis of N‐ACE FI structure correspond to two molecules in the asymmetric unit

	C‐ACE FI	N‐ACE FI	AnCE FI	AnCE FII
Station	DLS I02	DLS I03	DLS I04‐1	DLS I04
Mol. /AU	1	2	1	1
Resolution (Å)	1.85	1.91	1.82	1.99
Space group	*P2*_*1*_ *2*_*1*_ *2*_*1*_	*P1*	*R*3	*R*3
Cell dimension (Å, °)	*a* = 56.4, *b* = 85.0, *c* = 133.9 α = β = γ = 90.0	*a* = 72.9, *b* = 76.6, *c* = 82.5 α = 88.6, β = 64.2, γ = 75.6	*a* = *b* = 173.7, *c* = 102.2 α = β = 90, γ = 120	*a* = *b* = 172.9, *c* = 100.5 α = β = 90, γ = 120
Total no. of observations	210 408	398 681	573 835	191 609
No. of unique reflections	52 682	115 256	102 293	74 339
Completeness (%)	94.9 (94.4)	96.4 (90.4)	99.3 (96.7)	96.8 (95.4)
*I/*σ(*I*)	9.9 (2.4)	12.2 (2.1)	15.1 (2.5)	6.7 (1.5)
*R*_symm_^a^	9.2 (41.9)	6.3 (62.9)	6.7 (66.4)	9.8 (59.7)
*R*_cryst_^b^ */R*_free_^c^	18.2/21.3	18.8/22.2	17.7/19.4	19.5/21.6
Rmsd				
Bond lengths (Å)	0.008	0.008	0.008	0.008
Bond angles (°)	1.216	1.350	1.178	1.193
*B*‐factor analysis				
Protein all atoms	17.9	26.7/31.4	27.7	29.4
Protein main chain	17.2	25.9/30.6	27.0	28.7
Protein side chain	18.5	27.5/32.2	28.4	30.1
Inhibitor atoms	13.0	25.4/24.4	26.5	32.3
Zn^2+^ ion	10.3	18.0/16.6	24.1	24.2
Glycosylated sugars	38.8	53.0/64.4	48.5	59.1
Solvent atoms	23.6	31.7	35.9	34.3
PDB code	4ca5	4ca6	4ca7	4ca8

### Binding of FI with C‐ACE

**Figure 2:**
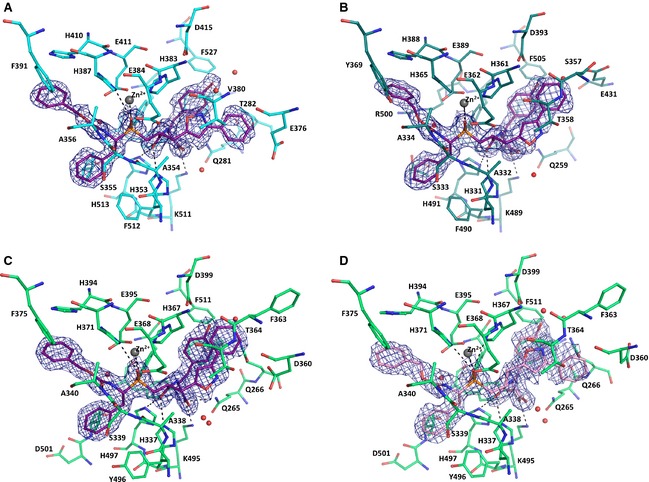
Binding of the dual inhibitors to angiotensin‐I converting enzymes: (A) FI (purple) bound to C‐ACE (cyan); (B) FI (purple) bound to N‐ACE (teal); (C) FI (purple) bound to AnCE (green); (D) FII (pink) bound to AnCE (green). Residues involved in inhibitor binding are shown as sticks; the zinc ion is shown as a grey sphere and waters in red. The omit map of the ligand is shown in blue density corresponding to the weighted difference map calculated without the ligand in refmac5 56 and displayed at 1σ level. Important hydrogen bonds (listed in Table 2) are indicated as dashed lines and also include the zinc coordination with the ligand.

The ACE/ECE‐1 FI dual inhibitor is a competitive inhibitor of both N‐ACE and C‐ACE. However, it is ~ 440‐fold more selective for C‐ACE (apparent *K*_i_ = 0.41 ± 0.03 nm 44). One bound inhibitor molecule was modelled in the catalytic site of C‐ACE (Fig. 2A) with the aid of the unambiguous electron density map at 1.85 Å.

**Figure 3:**
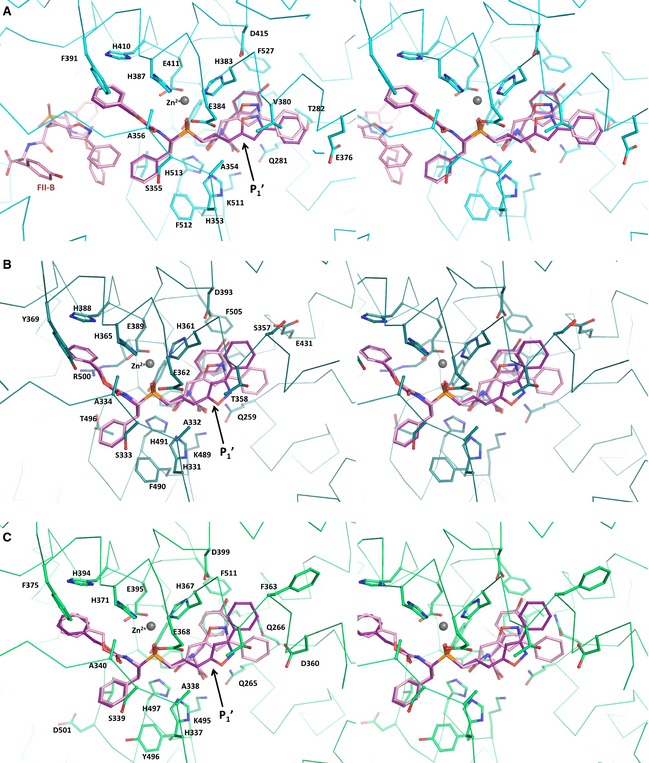
Comparison between the stereoisomers FI and FII binding to angiotensin‐I converting enzymes: (A) FI (purple) and FII (pink, PDB 2XY9 44) bound to C‐ACE (cyan); (B) FI and FII (PDB 2XYD 44) bound to N‐ACE (teal); (C) FI and FII bound to AnCE (green). Stereo representations of the structures in complex with FI and FII for each protein (aligned in pymol, all atoms superposition) and residues shown are from the enzymes in their respective complexes with FI only. The arrow indicates the location of the P_1_′ site of the ligand to highlight the difference between FI and FII.

As in the C‐ACE with FII structure 44, FI occupies all four substrate binding subsites (S_2_, S_1_, S_1_′ and S_2_′) and two phosphinic oxygen atoms (OAG and OAD) of the inhibitor make direct coordination with the catalytic zinc ion (distance 2.2 and 2.4 Å respectively) at the active site. In addition, it is held by 12 hydrogen bonds including four hydrogen bonds mediated through water molecules, as calculated by hbplus (Table 2). Similarly to the FII inhibitor, the phenyl moiety at the P_2_ position is held by aromatic interactions with Phe375 and His410 and a hydrogen bond between the carbonyl oxygen of the inhibitor and the main chain nitrogen atom of Ala356. The main chain amide nitrogen between the two phenyl moieties of FI is held via a water molecule to Arg522, Tyr523 and Glu411 which was also observed in the FII complex. The second phenyl group of FI at the P_1_ position is stabilized by hydrophobic interactions with residues Val518 and Phe512, similarly to what was observed with FII (Fig. 3A). The phosphinic oxygen atoms of FI are further anchored at the catalytic site through direct hydrogen interactions with His383 and His387 and the hydroxyl group of Tyr523. The P_2_' C‐terminal tyrosine moiety of FI interacts via a water molecule between its OH atom and Lys454 and appears to mediate hydrophobic interactions with aromatic residues Phe457 and Phe527 (Fig. 2A). This P_2_' position is further stabilized through a conserved mechanism of inhibition at the carboxy terminus 44 with strong hydrogen bonding to Lys511 and Tyr520, as well as the solvent‐mediated interactions with the S_2_′ site. The bulky side chain at P_1_' was clearly visible in its *S* conformation in FI. The isoxazole group appears to make a water‐mediated bond with the backbone of Val380 (Fig. 2A). Surprisingly, the isoxazole group shows a similar orientation in FII (Fig. 3A). However, in FII this group is held closer to the catalytic site by the *R* configuration, thereby allowing for direct hydrogen bonds with His383. The P_1_' aromatic group is further stabilized at this position through its interaction with the large surrounding S_1_′ hydrophobic pocket composed of Val380 and Val379.

**Table 2:** Hydrogen bond contacts of ACE homologues with the dual inhibitors

N‐domain ACE						C‐domain ACE						AnCE					
FI			FII^a^			FI			FII^a^			FI			FII		
Protein atom	Inhibitor atom	Distance (Å)	Protein atom	Inhibitor atom	Distance (Å)	Protein atom	Inhibitor atom	Distance (Å)	Protein atom	Inhibitor atom	Distance (Å)	Protein atom	Inhibitor atom	Distance (Å)	Protein atom	Inhibitor atom	Distance (Å)
Q259 NE2	O	3.0	Q259 NE2	O	3.0	Q281 NE2	O	3.0	Q281 NE2	O	3.0	Q265 NE2	O	3.2			
H331 NE2	OAC	2.7	H331 NE2	OAC	2.6	H353 NE2	OAC	2.7	H353 NE2	OAC	2.6	H337 NE2	OAC	2.3	H337 NE2	OAC	2.7
A334 N	OAB	3.0	A334 N T358 OG1	OAB NBG	2.8 3.45	A356 N H387 NE2	OAB OAD	3.0 3.1	A356 N H383 NE2 H387 NE2	OAB OAG OAG	2.9 3.1 3.1	A340 N H371 NE2	OAB OAD	2.9 3.1	A340 N H367 NE2 H371 NE2	OAB OAG OAG	2.8 3.2 3.1
K489 NZ	O	2.7	K489 NZ	O	2.6	K511 NZ	O	2.8	K511 NZ	O	2.7	K495 NZ	O	2.7	K495 NZ	O	2.9
H491 NE2	OAC	3.0	H491 NE2	OAC	3.0	H513 NE2	OAC	2.9	H513 NE2	OAC	2.8	H497 NE2	OAC	3.0	H497 NE2	OAC	3.0
Y498 OH	O	2.6	Y498 OH	O	2.6	Y520 OH	O	2.6	Y520 OH	O	2.6	Y504 OH	O	2.6	Y504 OH	O	2.6
Y501 OH	OAG	2.7	Y501 OH	OAD	2.5	Y523 OH	OAG	2.6	Y523 OH	OAD	2.6	Y507 OH	OAG	2.5	Y507 OH	OAD	2.5
Wat	NBI	3.0	Wat	NBI	3.0	Wat Wat	NBI NBG	3.1 2.8	Wat Wat Wat	NBI NBG OBK	3.1 2.8 3.4	Wat Wat Wat	NBI OBK OXT	3.0 2.7 3.1	Wat Wat	NBI OXT	3.0 3.0
Wat	OXT	2.8	Wat	OXT	2.7	Wat	OXT	2.7	Wat	OXT	2.7	Wat	OXT	2.8	Wat	OXT	2.8
Wat	OH	2.7	Wat	OH	2.4	Wat	OH	2.7	Wat Wat Wat	OH OH O	2.8 2.8 3.5	Wat	OH	2.8	Wat Wat	OH OH	2.8 3.3

a From Akif *et al*. 44.

Overall, FI and FII are both strong inhibitors of C‐ACE with similar potency (*K*_i_ = 0.41 ± 0.03 and 0.61 ± 0.03 nm, respectively 44). The overall binding mechanism has some common features with other phosphinic inhibitors 28 but shows key differences at the P_1_' site, with C‐ACE accommodating the *S* configuration of FI through hydrophobic interactions with the S_1_' cavity. The surprising feature of FII was its novel secondary binding site in C‐ACE 44. This was made feasible by a series of hydrophobic interactions with C‐ACE's allosteric site, and also importantly via a ‘hand‐shake’ interaction between two molecules of the same inhibitor, with P_2_ of the active‐site‐bound FII making π−π stacking interaction with the isoxazole phenyl of the secondary FII (Fig. 3A). This phenomenon was not visible here with FI. Although the configurations of FI and FII are similar at their P_2_ site, the *S* orientation of FI prevents the stacking interaction as the secondary molecule would clash with the inhibitor at the primary binding site (hence it is unlikely to be accommodated due to steric hindrance). It is interesting to note that this major difference does not result in a higher *K*_i_ for FII. This would suggest that FII binding at the secondary site is probably a consequential event requiring binding of the inhibitor at the primary (catalytic) site. Further binding experiments may clarify the importance of the allosteric binding site of FII and help to design a novel inhibitor targeting that area that would focus on preventing substrate binding 45. However, inhibition of the catalytic efficiency in this case is probably dependent on the primary site.

### Binding of FI with N‐ACE

The crystal structure of N‐ACE was solved in complex with FI at 1.9 Å. A single molecule of FI was fitted in the electron density map at the active site (Fig. 2B). The overall binding mode was similar to that described for FII 44 with the tripeptidic backbone of FI and FII superposing well (Fig. 3B). The main anchor is the coordination of the catalytic zinc ion with the phosphinic oxygen atoms of FI (OAG and OAD, at a distance of 2.1 and 2.5 Å, respectively). Additional binding strength is provided by 10 hydrogen bonds, including three water‐mediated interactions with the protein (Table 2). As expected, the only difference in the binding of the two stereoisomers resides at the P_1_' position. The isoxazole group of FI appears unable to make the weak hydrogen bond seen between FII and Thr358 (Table 2) because of the 180° rotation of the side chain. The P_1_' bulky side chain fits within the large N‐ACE S_1_′ cavity but seems to provide limited interaction with the protein. It forms a stacking arrangement with the tyrosine group at P_2_′ which is itself surrounded by aromatic residues (Phe435 and Phe505) and forms an anchoring point with its C‐terminal oxygen atoms strongly binding (four hydrogen bonds) with the S_2_′ site. This subtle difference in binding at the P1′ due to the *S* and *R* configurations may explain the slight difference in *K*_i_ between FI and FII (180 ± 25 against 150 ± 20 nm, respectively 44).

### Binding of the dual inhibitors FI and FII to *Drosophila* ACE

**Figure 4:**
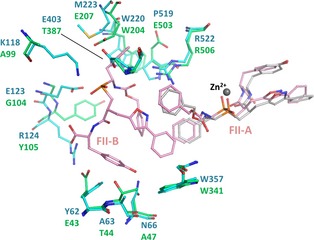
Comparison between the secondary binding site of FII in C‐ACE and the corresponding site in AnCE: FII‐A and FII‐B (pink, PDB 2XY9 44) bound to C‐ACE (cyan) at the primary and secondary sites, respectively; FII (grey) bound to AnCE (green). Only residues involved in the binding of FII‐B in C‐ACE and the corresponding residues in AnCE are shown.

Both stereoisomers of the dual inhibitor bind to AnCE with apparent *K*_i_ values of 24 and 120 nm for FI and FII respectively. The crystal structures of AnCE in complex with FI and FII were solved at 1.8 and 2.0 Å respectively. Each inhibitor could be fitted at the catalytic site of the enzyme and interacted with the zinc ion through direct coordination (Fig. 2C,D). The position of the inhibitors in AnCE is similar to that observed in their respective human homologues 44. It resides within the S_2_ to S_2_′ channel through multiple interactions, including 13 hydrogen bonds, most of them conserved through the ACE–dual inhibitor complexes (Table 2). The P_2_′ position is again well anchored within the S_2_′ pocket not only through direct hydrogen bonds of its carboxy‐terminal oxygen atoms but also with the help of a strong network of solvent molecules observed at this site. The conserved hydrophobic patch made of Phe441 and Phe511 in AnCE also allows for aromatic interactions with the tyrosine side chain of FI and FII (Fig. 3C). The P_1_′ configuration adopted by FI and FII in AnCE is similar to that observed with N‐ACE (Fig. 3C). With FI, the isoxazole group is closer to the P_2_′ pocket and interacts with the solvent network while in FII it is in proximity (3.6 Å) to the zinc coordinating residue His367. The phenyl moiety in FI forms a parallel stacking with the tyrosine group at P_2_′ while in FII it is located deeper within the catalytic channel and causes a shift of the Asp360 side chain (Fig. 2C,D). The stronger interactions of FI at the P_1_′ site may explain the better inhibition of AnCE by FI over FII. In the non‐prime binding site, S_1_ and S_2_ residues are well conserved between human and the *Drosophila* ACE and thus allow for the hydrophobic interactions governing the binding of the dual inhibitors at these sites, in particular between the P_2_ phenyl group and His371, Phe375 and His394 (Fig. 2). A comparison of FI and FII in the obligatory binding site of AnCE and C‐ACE structures shows similar orientation of the inhibitor molecule by retaining the conserved hydrogen bond interactions at the catalytic site. The two phenyl moieties of the dual inhibitor at the P_1_ and P_2_ position are identically placed in the two structures and their positions are also conserved in the previously described C‐ACE specific phosphinic inhibitor RXPA380 in complex with AnCE and C‐ACE 28. It should be added that the P_2_ site in FII with AnCE appears in a slightly different conformation to that in FI but does not influence the overall binding (Fig. 4).

A comparison of AnCE and C‐ACE 44 with respect to the secondary FII‐B binding site shows that Glu403, Glu123 and Lys118 in C‐ACE hold the second molecule of the dual inhibitor by making direct interactions with the phosphinic oxygen atom; however, these residues are replaced by shorter side chain residues in AnCE – Thr387, Gly104 and Ala99, respectively. Interestingly, the presence of Glu207 and Glu503 in AnCE occludes the pockets that would accommodate the phenyl moieties of FII‐B. In addition, the orientation of the tyrosine ring of FII‐B in AnCE would cause steric hindrance with Thr44 and Glu43, and these residues are replaced by Ala63 and Tyr62 at equivalent positions in the C‐ACE inhibitor complex 44. Furthermore, Asn66, which holds the tyrosine moiety of FII‐B through hydrogen bond interaction in C‐ACE, is replaced with Ala47 in the AnCE structure, therefore losing this interaction (Fig. 4).

## Discussion

### Overall binding (FI and FII)

**Figure 5:**
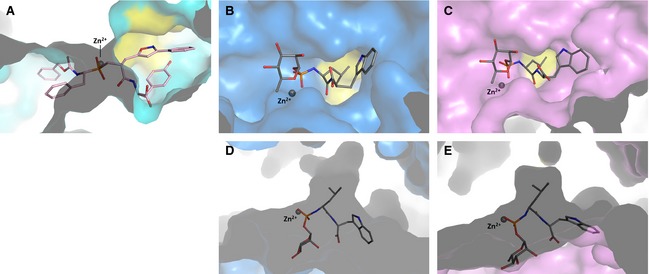
Phosphinic inhibitor binding at the active site of C‐ACE, ECE‐1 and neprilysin: (A) FII (pink, PDB 2XY9 44) bound to C‐ACE (cyan); (B), (D) phosphoramidon (grey) bound to ECE‐1 (blue, PDB 3DWB 46); (C), (E) phosphoramidon (grey) bound to neprilysin (pink, PDB 1DMT 47). The catalytic channel of each protein is represented in surface mode with its respective S_1_′ subsite highlighted in yellow. The catalytic zinc ion is shown as a grey sphere.

The structures presented here of FI bound to the three homologous ACE proteins and FII bound to AnCE complete our understanding of the binding mechanisms of these inhibitors. Despite their difference in chirality, FI and FII show similar behaviour on ACE in terms of inhibition and domain specificity; indeed both present sub‐nanomolar *K*_i_ and at least 200‐fold specificity for C‐ACE. These similarities may be explained by a common set of interactions. First the phosphinic group offers a strong binding to the catalytic zinc ion and the phenyl groups at P_2_ and P_1_ present conserved hydrophobic interactions at the S_2_ and S_1_ site respectively. The carboxyl termini of FI and FII are held in position by strong hydrogen bonds and peptidomimetic interactions seen with angiotensin II bound to C‐ACE, namely Gln281, Lys511 and Tyr520 45. The P_2_′ tyrosine residue of the inhibitors enhances binding at the S_2_′ subsite through aromatic interactions with ACE. ACE is quite unusual compared with other zinc proteases (Fig. 5) in that it shows a fairly large cavity downstream of the catalytic channel which can accommodate bulky groups at the P_1_′ site. This is highlighted by the capacity of both isomers of the dual inhibitor to bind ACE despite the large isoxazole phenyl side group. The higher specificity for C‐ACE is probably due to the more hydrophobic nature of this domain's active site compared with N‐ACE (Table 3) which confers tighter binding for the aromatic moieties of the inhibitor.

**Table 3:** Sequence variation of ACE residues involved in hydrophobic interactions with FI and FII

Residues			Position
C‐ACE	N‐ACE	AnCE	
H387	H365	H371	S_2_
V379	S357	F363	S_1_′
V380	T358	T364	S_1_′
F391	Y369	F375	S_2_
H411	H388	H394	S_2_
F457	F435	F441	S_2_′
V518	T496	V502	S_1_
F527	F505	F511	S_2_′

### Specific observations on FI binding

The difference between FI and FII resides in the orientation of the isoxazole phenyl group at P_1_′ and is a consequence of the *S* or *R* conformation. It is interesting to observe that this group seems to have itself a degree of flexibility, particularly in FI. The oxygen atom (OBK) of the isoxazole in the C‐ACE complex (Fig. 2A) is oriented towards the catalytic site whereas it is seen in the opposite direction with N‐ACE and AnCE. This also results in the phenyl group being deeper in the catalytic channel in C‐ACE. The surprising observation from the structures of FI with the ACE homologues is that the active site of these enzymes does not seem to change upon ligand binding, with little variation in the position of the residues involved with root‐mean‐square deviation (rmsd) values of < 0.3 and < 0.7 Å for all main chains and side chain atoms, respectively, compared with the ligand‐free enzymes (Table 4). On the contrary, it is the ligand itself that adapts to the exact shape of the catalytic pocket for each of the domains.

**Table 4:** Comparison of active site residues upon ligand binding. The ligand‐free, FI‐bound and FII‐bound enzymes were superposed using the Superpose routine of the ccp4 program suite 54 and the rmsd values of all atoms within 15 Å radius of the active site residues were calculated. PDB codes 1o8a (ligand‐free) and 2xy9 (FII‐bound) used for C‐ACE; 2c6f (ligand‐free) and 2xyd (FII‐bound) for N‐ACE; 2x8y for ligand‐free AnCE

	Rmsd of all atoms for main chain/side chain (Å)		
	Ligand‐free vs. FI‐bound	Ligand‐free vs. FII‐bound	FI‐bound vs. FII‐bound
C‐ACE	0.141/0.383	0.206/0.457	0.160/0.261
N‐ACE	0.281/0.641	0.291/0.677	0.112/0.211
AnCE	0.107/0.315	0.098/0.339	0.103/0.204

### Specific observations on FII binding

The positioning of FII at the primary binding site (A) appears consistent in all three ACE homologues. The peculiar binding mode of FII in C‐ACE, where a secondary molecule (B) was unexpectedly bound 44, was not observed with FI. This is a direct result of the *S* configuration which prevents the stacking interaction between P_1_′ of B and P_2_ of A. Interestingly FI and FII share similar *K*_i_ for C‐ACE and thus the presence of FII‐B at the allosteric site does not seem to influence the inhibition potency. It would also suggest that the presence of FII‐B is only possible through interaction with FII‐A. The catalytic channel is known to show flexibility in accommodating larger ligands. The crystal structure of C‐ACE in complex with the bradykinin potentiating peptide (BPPb) demonstrated this capacity through movement of the helices capping the channel 45. Noticeably, FII‐B occupies the same pocket as the N‐terminus of the BPPb peptide. This secondary site might therefore be an attractive target for allosteric ligands that would prevent substrate binding. However, this approach would focus on conferring ACE‐domain specificity rather than the multi‐enzyme inhibition presented here.

### Specificity of the dual inhibition for ACE and ECE‐1

The importance of the inhibitor's chirality is highlighted when looking at its specificity towards multiple proteases. Indeed FI possesses sub‐micromolar *K*_i_ for ACE, ECE‐1 and neprilysin 35. On the other hand, FII is a strong inhibitor of ACE (*K*_i_ = 0.65 nm) and ECE‐1 (*K*_i_ = 14 nm) but a weak inhibitor of neprilysin (*K*_i_ = 6.7 μm) 44. This difference in inhibition may be linked to the structural constraints given by the S_1_′ subsite of the aforementioned enzymes. The structures of ECE‐1 and neprilysin have been solved in complex with the phosphinic metalloprotease inhibitor phosphoramidon 46. These structures have in common a direct coordination of the zinc ion through the phosphinic group and allow a comparison with the dual inhibitor bound to ACE (Fig. 5). Phosphoramidon presents a classical *S* configuration which permits accommodation of the P_1_′ leucine to fit within the S_1_′ narrow pocket in both ECE‐1 (Fig. 5B,D) and neprilysin (Fig. 5C,E). A more recent structure of neprilysin in complex with a novel inhibitor (MCB3937 48) also demonstrated that neprilysin could accommodate a phenylalanine at this position. Considering the inhibition constants of FI and the structural data available, it is expected that the S_1_′ hydrophobic pocket would be able to fit the bulkier isoxazole phenyl group of the *S* inhibitor. However, FII in its *R* configuration would most probably clash with the residues at the entrance of S_1_′ and the zinc coordinating site (Fig. 5E). ECE‐1 on the contrary shows a deeper S_1_′ pocket (Fig. 5D) that should allow the inhibitor to bind in either configuration. Further structural work with ECE‐1 and the inhibitor should help refine the specificity for both target enzymes, ACE and ECE‐1.

## Conclusion

The structures of ACE (N‐ and C‐ACE) and AnCE in complex with the phosphinic inhibitors FI and FII provide a comprehensive picture of their binding mechanisms. The difference in chirality at the P_1_′ group confers FII (*R* configuration) selectivity towards the target enzymes ACE and ECE‐1. Interestingly, both enantiomers (FI has an *S* configuration) show a similar degree of inhibition and specificity towards the three homologous ACE domains presented here. The comparison between these structures has highlighted subtle differences in the binding mode of these inhibitors; more particularly it has highlighted how the ligand itself can adapt to fit within the catalytic pocket. The originality of FII molecule binding at two sites within C‐ACE was confirmed to be specific to this enantiomer and only the C‐domain. With FI and FII showing similar inhibition of C‐ACE, it can be inferred that the binding of the secondary FII at the allosteric site does not affect the efficiency of inhibition. This site, however, may be considered a novel target for allosteric domain‐specific inhibition of ACE that would prevent substrate binding. The molecular details of the interactions of FI and FII at the S_1_′ subsite have provided extensive information to understand how a difference in chirality may provide varied specificity. The surprising flexibility of FI also demonstrates the difficulty in predicting these types of ligand‐binding interactions and thus emphasizes the need for high resolution structural information. These observations will have implications in refining the domain specificity in human ACE and for the development of highly selective non‐peptide inhibitors with multiple targets of therapeutic interest.

## Materials and methods

### Synthesis of the phosphinic tripeptide inhibitors FI and FII

The phosphinic tripeptide inhibitors FI and FII, (2*S*)‐2‐({3‐[hydroxyl (2‐phenyl‐(1*R*)‐1‐{[(benzyloxy) carbonyl]‐amino}ethyl)phosphinyl]‐2‐[(3‐phenylisoxazol‐5‐yl)methyl]‐1‐oxo‐propyl}amino)‐3‐(4‐hydroxy‐phenyl) propanoic acid, were synthesized as recently reported 35.

### Expression and purification of proteins

#### C‐domain (testis) ACE

A variant of human testis ACE (tACEΔ36‐g13, underglycosylated protein) was purified to homogeneity from CHO (Chinese‐hamster ovary) cells, as described previously 35. Crystals of C‐ACE in complex with FI were grown at 16 °C by the hanging‐drop vapour diffusion method. C‐ACE protein (10 mg·mL^−1^ in 50 mm HEPES, pH 7.5) was pre‐incubated with FI (2 mm) on ice for 2 h before crystallization. The pre‐incubated sample (2 μL) was mixed with the reservoir solution, consisting of 15% PEG [poly(ethylene glycol)] 3350, 100 mm MIB (sodium malonate, imidazole, and boric acid; pH 4.0), 5% glycerol and 10 μm ZnSO_4_, and suspended above the well. Diffraction quality co‐crystals appeared after ~ 1 week.

#### N‐domain ACE

The minimally glycosylated construct of the N‐domain of somatic ACE, N‐ACE389, was generated by site‐directed mutagenesis, as described previously 43. The recombinant protein was expressed in CHO cells and purified to homogeneity by lisinopril affinity chromatography. The crystals of N‐ACE in complex with FI were grown at 16 °C by the hanging‐drop method. N‐ACE protein (5 mg·mL^−1^) was pre‐incubated with FI (2 mm) on ice before crystallization; 2 μL of pre‐incubated sample was mixed with an equal volume of 60 mm divalent cations (Molecular Dimensions, Newmarket, UK), 0.1 m Tris/Bicine (pH 8.5) and 30% PEG 550MME/PEG 20000 (Molecular Dimensions, Newmarket, UK). Crystals appeared within 48 h.

#### AnCE

AnCE was cloned and expressed in *Pichia pastoris* as described previously 49. The purified protein was co‐crystallized with the inhibitors by pre‐incubating on ice for 2 h the protein (10 mg·mL^−1^ in 50 mm HEPES pH 7.5, 0.1 mm phenylmethanesulfonyl fluoride and 10 μm zinc acetate) with FI (2 mm) and FII (2 mm) respectively. Crystals of the complexes were obtained with 2 μL of pre‐incubated protein‐inhibitor sample mixed with an equal volume of reservoir solution (100 mm HEPES pH 7.5 and 1.3 m sodium citrate) and suspended above the well as a hanging drop. Diffraction quality co‐crystals of the complexes appeared after about 1 week.

AnCE activity was assayed using hippuryl‐histidyl‐leucine as the substrate (*K*_m_ 1.468 mm, standard error 0.178 mm) and 5 ng of enzyme stabilized with 2 μg of bovine serum albumin per assay, according to the method previously described 50. Inhibitors were pre‐incubated with AnCE for 10 min before addition of substrate. The *K*_i_ for the inhibition of recombinant AnCE with the dual inhibitors FI and FII was estimated from the IC_50_ values obtained using graphpad prism (FI, 44 nm, 95% confidence interval 34–58 nm; FII, 210 nm, 95% confidence interval 180–246 nm) using the method of Cheng and Prusoff 51.

### X‐ray diffraction and data processing

X‐ray diffraction data for C‐ACE+FI, N‐ACE+FI, AnCE+FI and AnCE+FII complexes were collected on several MX stations at the Diamond Light Source (Oxon, UK; Table 1). Crystals of C‐ACE+FI were cryoprotected with 35% PEG 3350; no cryoprotectant was used to keep the other crystals at constant temperature (100 K) under the liquid nitrogen jet during data collection. Raw data images were indexed and integrated with mosflm 52 or xds 53. Data reduction was carried out by using the ccp4 program scala 54. Initial phases for structure solution were obtained using the molecular replacement routines of the phaser program 55. The atomic coordinates of C‐ACE (PDB code 1O8A 26), N‐ACE (PDB code 2XYD 43) and AnCE (PDB code 2X8Y, 42) were used as a search model for their respective complexes. The resultant solutions were refined using refmac5 56 and adjustment of the models was carried out using coot 57. Water molecules were added at positions where *F*_o_ − *F*_c_ electron density peaks exceeded 3σ and potential hydrogen bonds could be made. Based on electron density interpretation, the inhibitor and sugar moieties were added in the complex structures and further refinement was carried out. The coordinates and parameter files for the dual inhibitor were generated using the prodrg server 58. Validation was conducted with molprobity 59. Figures were drawn with pymol (DeLano Scientific, San Carlos, CA, USA). Hydrogen bonds were verified with the programs hbplus 60 and ligplot 61. The detailed refinement statistics for the complex structure are given in Table 1. The atomic coordinates and the structure factors have been deposited with the RCSB Protein Data Bank under the codes 4ca5, 4ca6, 4ca7 and 4ca8.
